# Similar DNA methylation levels in specific imprinting control regions in children conceived with and without assisted reproductive technology: a cross-sectional study

**DOI:** 10.1186/1471-2431-12-33

**Published:** 2012-03-20

**Authors:** Susan E Puumala, Heather H Nelson, Julie A Ross, Ruby HN Nguyen, Mark A Damario, Logan G Spector

**Affiliations:** 1 Center for Health Outcomes and Prevention Research, Sanford Research, Sioux Falls, SD, USA; 2 Department of Pediatrics, Sanford School of Medicine of the University of South Dakota, Sioux Falls, SD, USA; 3 Division of Epidemiology and Community Health, School of Public Health, University of Minnesota, Minneapolis, MN, USA; 4 Masonic Cancer Center, University of Minnesota, Minneapolis, MN, USA; 5 Division of Epidemiology/Clinical Research, Department of Pediatrics, University of Minnesota Medical School, Minneapolis, MN, USA; 6 Department of Obstetrics, Gynecology and Women's Health, University of Minnesota Medical School, Minneapolis, MN, USA

**Keywords:** Assisted reproductive technology, Epigenetics, Imprinting

## Abstract

**Background:**

While a possible link between assisted reproductive technology (ART) and rare imprinting disorders has been found, it is not clear if this is indicative of subtler disruptions of epigenetic mechanisms. Results from previous studies have been mixed, but some methylation differences have been observed.

**Methods:**

Children conceived through ART and children conceived spontaneously were recruited for this cross-sectional study. Information about reproductive history, demographic factors, birth characteristics, and infertility treatment was obtained from maternal interview and medical records. Peripheral blood lymphocytes and buccal cell samples were collected from participating children. Methylation analysis was performed on five loci using pyrosequencing. Statistical analysis of methylation differences was performed using linear regression with generalized estimating equations. Results are reported as differences with 95% confidence intervals (CI).

**Results:**

A total of 67 ART children and 31 spontaneously conceived (SC) children participated. No significant difference in methylation in lymphocyte samples was observed between groups for any loci. Possible differences were found in buccal cell samples for *IGF2 DMR0 *(Difference: 2.07; 95% confidence interval (CI): -0.28, 4.42; *p *= 0.08) and *IGF2R *(Difference: -2.79; 95% CI: -5.74, 0.16; *p *= 0.06). Subgroup analysis indicated potential lower methylation in those whose parents used ART for unexplained infertility.

**Conclusions:**

Observed differences in methylation between the ART and SC groups were small for all loci in the two sample types examined and no statistical differences were observed. It is still unclear whether or not small differences observed in several studies represent a real difference between groups and if this difference is biologically meaningful. Larger studies with long term follow-up are needed to fully answer these questions.

## Background

Use of assisted reproductive technology (ART), including *in vitro *fertilization (IVF) and intracytoplasmic sperm injection (ICSI), is rapidly rising in the United States (US) and curxxrently accounts for over one percent of all infants born each year [1]. The potential for epigenetic disruptions in children born after infertility treatment, particularly ART, was first seen in a number of studies showing an increase in imprinting disorders such as Beckwith-Wiedemann syndrome (BWS), Angelman syndrome (AS), and Silver-Russell syndrome (SRS) in these children [2-16]. These studies further indicated that the cause of the imprinting disorders was due to epigenetic disruptions rather than mutations or uniparental disomy [4,5,7,9-11,13,17-20].

Imprinting disorders are very rare and, even with a relative increase in incidence of these disorders, most children conceived through ART are healthy. However, a relative increase suggests the possibility for more frequent, but subtler disruptions of epigenetic mechanisms. It has been suggested that such epigenetic disruptions could potentially manifest themselves as an increased propensity for childhood cancer as well as adult onset diseases such as cancer and heart disease that are thought to be epigenetically mediated [21,22].

Several recent studies have examined differences in methylation in various imprinted gene regions after ART in peripheral blood, placenta tissue, buccal cells, cord blood, chorionic villus samples, and embryos [23-38]. Results have been mixed and difficult to synthesize due to differences in gene regions, tissues examined, and ways of assessing DNA methylation. However, some studies have indicated a difference in DNA methylation or gene expression in the various gene regions [24,26,27,30,32,33], although other studies have not observed a difference [25,28,29,31,34-37].

Given the mixed evidence so far, and since genes in the 11p15 region and the *IGF2R *gene located at 6q26 have been associated with BWS and SRS and many different types of cancer, we were interested in further exploring these regions for differential methylation. To assess quantitative DNA methylation differences between in children conceived after ART treatment and children conceived spontaneously, we conducted a cross-sectional study and focused on peripheral blood and buccal cell samples. Specifically, we examined quantitative methylation values at the 11p15 region including two CTCF binding sites within *H19*, one differentially methylated region (DMR) in *IGF2*, and the imprinting control region *KvDMR *as well as a DMR in the *IGF2R *gene located at 6q26. Although some of these sites have been commonly examined (e.g. *KvDMR *and CTCF binding sites in *H19*), little information is available about methylation differences in the *IGF2 DMR0 *and *IGF2R *regions each having been explored in only one prior study [33,38].

## Methods

### Study population

Two groups of children were recruited for this study; one conceived through ART (ART group) and the other born after spontaneous conception (SC group). ART children had to be conceived through IVF or IVF + ICSI with fresh non-donor oocytes. SC children had to have been conceived without the use of any fertility drugs or treatments. In the case of multiple births in either the ART or SC group, only one child was selected for participation in the study. Children diagnosed with BWS, AS, Prader-Willi syndrome, or retinoblastoma were excluded from the study.

ART children and their mothers were recruited from the University of Minnesota Reproductive Medicine Center. Mothers who conceived through ART and reported a live birth between March 2005 and December 2008 were contacted about participation of their child through a letter and follow-up phone call. Out of 328 women identified as potentially eligible, 99 agreed to participate and 67 children completed the clinic visit.

SC children were recruited through advertisements posted in and around the University of Minnesota. Similar to the ART children, SC children had to have been born between March 2005 and December 2008. A total of 45 women agreed to participate in the study and 31 children completed the clinic visit prior to the close of the study.

This study was approved by the institutional review board at the University of Minnesota. All mothers provided informed consent for themselves and their child prior to participating in the study.

### Sample collection

Peripheral blood lymphocytes and buccal cell samples were collected either at a research clinic or at the participant's local clinic. Samples from the research clinic were delivered for processing immediately, while other clinic samples were sent express mail to Dr. Nelson's laboratory and were processed immediately upon receipt (usually within 24 h of sample collection). Up to 6 ml of blood was collected from each child through venipuncture. Buccal cells were collected using Catch-All™ Sample Collection Swabs (Epicentre, Madison, WI, USA). Two swabs were collected from each child.

### Data collection

Information about ART and SC mothers and children was collected through a brief questionnaire as well as from delivery records. In addition, for ART mothers, information on specific procedures was obtained from medical records. The questionnaire collected demographic information, as well as maternal reproductive history and the participating child's birth characteristics. All maternal and child characteristics used in the analysis apart from birth weight were based on questionnaire data. Birth weight was taken from the delivery records. If birth weight was not available on the birth record, maternal report from the study questionnaire was used. SC mothers were also asked about time to pregnancy to assess possible infertility that resolved without treatment. Information obtained from infertility clinic records included indication for infertility, number of cycles of treatment, and specific procedure information.

### Genetic regions analyzed

Three regions of interest were examined for differential methylation: *IGF2/H19, KvDMR*, and *IGF2R*. These regions were selected based on their association with BWS, SRS, and cancer. Specifically, we analyzed *IGF DMR0 *(3 CpGs), 3 rd (11 CpGs) and 6th (16 CpGs) CTCF-binding site of *H19 DMR, IGF2R *(15 CpGs), and *KvDMR *(7 CpGs). The 5th CpG cite in the 6th CTCF-binding site of *H19 DMR *is known to be polymorphic and so was excluded from the analysis [39].

### Methylation analysis

Methylation analysis was performed using pyrosequencing. Genomic DNA was isolated from the lymphocyte and buccal cell samples and treated with sodium bisulfite using the EZ methylation kit (Zymo research). This treatment actively changes unmethylated cytosines to uracils while leaving methylated cytosines unchanged. Primers and procedures for *IGF DMR0*, 3^rd ^and 6^th ^CTCF-binding site of *H19 DMR, IGF2R *were the same as in Boissonnas, et al. [39]. The protocol for the *KvDMR *region was different and based on Bourque, et al. [40]. After PCR amplification, pyrosequencing was performed for all regions using the PyroMark MD system, and analyzed using the accompanying software (Qiagen, Germantown/Gaithersburg, USA).

The percent methylation for each CpG was calculated by taking the peak height of the methylated cytosines divided by the sum of the peak height of the methylated and unmethylated cytosine. Several quality assurance tests were performed to assess the sequence generated by the pyrosequencing reaction against the expected sequence. CpG sites that consistently failed quality assurance tests were excluded from the analysis. We excluded one CpG site from *H19 CTCF3*, one CpG site from *H19 CTCF6 *and six CpG sites from *IGF2R *due to quality and polymorphism issues. Two children were excluded in locus-specific analysis due to unexpected sequences, likely due to polymorphisms, one in the *KvDMR *locus and the other in the *IGF2 DMR0 *locus.

To assess the reliability of the methylation assays, we obtained lymphocyte and buccal cell samples on nine healthy adults. Samples were split and processed on different days for three loci (*KvDMR, H19 CTCF3*, and *IGF2R*). Reliability was assessed using an intraclass correlation coefficient (ICC). While there were a few low ICC values for individual CpG sites, most of the sites had high ICC values (Additional file 2). We found that there was poor reliability for methylation analysis over amplifications for the *KvDMR *region in lymphocyte samples (ICC median (Interquartile range (IQR)): 0.54 (0.44-0.56)). However, this lower reliability was due to an overall lower methylation level for one of the three amplifications. Since a linear transformation of one of the three amplifications greatly improved reliability (ICC median (IQR): 0.78 (0.62-0.81)) accounting for amplification in our analysis will adjust our data for possible similar discrepancies. Reliability was goo d for the *KvDMR *region in buccal samples with all individual CpG sites found to have an ICC greater than 0.7 (ICC median (IQR): 0.87 (0.84-0.87)). The *H19 CTCF3 *region had good reliability for both buccal cells (ICC median (IQR): 0.73 (0.66-0.77)) and lymphocytes (ICC median (IQR): 0.76 (0.73-0.81)). The *IGF2R *region also had good reliability for both buccal cells (ICC median (IQR): 0.78 (0.59-0.81)) and lymphocytes (ICC median (IQR): 0.88 (0.70-0.89)).

### Data analysis

Descriptive data were compared between the two groups using Fisher's exact tests for categorical variables, and t-tests for continuous variables. Differences in methylation between groups were analyzed using linear models with generalized estimating equations (GEE). The GEE model accounts for the correlation between CpG cites within an individual. Each locus was considered separately. Adjusted models were constructed using variables related to use of ART: maternal age (continuous), maternal education (some college or less, college graduate, advanced degree), household income (< $40,000, $40,000-$79,999, ≥ $80,000) and child's birth weight (continuous); and variables possibly associated with methylation: child's age (continuous) and sex (male, female). All models also included a variable indicating day of the pyrosequencing run to control for any amplification effects. Subgroup analysis was performed within the ART group to examine differences in methylation by type of infertility (female only, male only, both male and female, unexplained). Results are reported as group means and 95% confidence intervals (CI) for the difference between groups. Sensitivity analysis was performed excluding the samples that were rerun after assay failure. All analysis was performed using SAS 9.2 (SAS Institute, Inc, Cary, NC).

## Results

A total of 67 children were included in the ART group and 31 in the SC group. Of these, 53 ART children and 27 SC children provided a blood sample while 67 ART children and 30 SC children provided a buccal cell sample. Demographic factors of the two groups are presented in Table 1. Many factors were different between the two groups, with the ART group tending to have higher household income, increased maternal age, and greater frequency of multiple births. Children in the ART group tended to be younger and have lower birth weights.

**Table 1:** Descriptive statistics by study group

Variable	ART group n = 67	SC group n = 31^a^	P-value
*Maternal Characteristics*	n (%)	n (%)	

Race			

White	64 (95.5)	27 (90.0)	0.37^b^

Non-white	3 (4.5)	3 (10.0)	

Education			

< College degree	10 (14.9)	10 (33.3)	0.10^b^

College degree	26 (38.8)	11 (36.7)	

Advanced degree	31 (46.3)	9 (30.0)	

Household income			

< 40 K	4 (6.0)	7 (23.3)	0.02^b^

40 K- < 80 K	18 (26.9)	10 (33.3)	

80 K +	45 (67.2)	13 (43.3)	

Age at child's birth			

Mean (SD)	34.1 (3.9)	29.6 (4.3)	< 0.001^c^

Time to pregnancy			

Not trying			9 (31.0)

< 12 months			18 (62.1)

≥ 12 months			2 (6.9)

*Child Characteristics*	n (%)	n (%)	

Sex			

Female	32 (47.8)	13 (43.3)	0.83^b^

Male	35 (52.2)	17 (56.7)	

Plurality			

Singleton	44 (65.7)	30 (100.0)	< 0.001^b^

Twins+	23 (34.3)	0 (0.0)	

Year of birth			

2005	7 (10.4)	9 (30.0)	0.11^b^

2006	23 (34.3)	8 (26.7)	

2007	23 (34.3)	10 (33.3)	

2008	14 (20.9)	3 (10.0)	

Birth weight (grams)			

mean (SD)	3005.57 (790.9)	3458.1 (701.9)	0.008^c^

Age (years)			

mean (SD)	2.5 (0.97)	3.0 (1.00)	0.02^c^

a One mother did not fill out the questionnaire and is missing all responses

b Fisher's exact test

c Two-sample *t*-test

Medical records for infertility treatment could not be obtained for six women, leaving 61 subjects for analysis of specific infertility diagnoses. Infertility diagnoses were fairly equally divided between female factor only (n = 21, 34%), male factor only (n = 17, 28%), and both male and female factors (n = 16, 26%). No medical explanation for infertility was found in 10% (n = 6) of couples and one couple was seeking treatment for reasons other than fertility. Most couples used ICSI for at least some of the embryos (84%) and over 70% had two embryos transferred.

No large differences in methylation were found between the ART and SC groups in either lymphocyte or buccal cell samples. Table 2 displays the average differences and 95% CI for the adjusted models. A possible, but statistically non-significant difference was seen in buccal cells only at *IGF2 DMR0*, with the ART group having higher methylation levels compared to the SC group (Difference: 2.07; 95% CI: -0.28, 4.42; *p *= 0.08). Larger, but non-significant, differences were also observed for the *IGF2R *region; for both buccal cells (Difference: -2.79; 95% CI: -5.74, 0.16; *p *= 0.06) and lymphocytes (Difference: -4.41; 95% CI: -9.49, 0.66; *p *= 0.09) there was an indication of lower levels of methylation in the ART group. Estimates were similar when limited to those samples that were not rerun due to assay failure (data not shown).

**Table 2:** Adjusted regression model results

Gene region of interest	ART group		SC group		Difference Estimate^c^	95% CI	p-value
	N^a ^	Mean^b^	N^a ^	Mean^b^			
Lymphocyte samples							

KvDMR	52	47.61	27	47.53	0.72	(-1.00, 2.45)	0.41

H19 CTCF3	53	42.16	27	41.70	0.66	(-0.94, 2.25)	0.42

H19 CTCF6	52	38.07	27	35.15	0.93	(-1.79, 3.65)	0.50

IGF2DMR0	53	50.04	26	48.32	0.08	(-1.82, 1.98)	0.94

IGF2R	52	69.26	27	72.13	-4.41	(-9.49, 0.66)	0.09

Buccal samples							

KvDMR	64	53.46	29	52.60	1.60	(-2.77, 5.98)	0.47

H19 CTCF3	65	42.94	29	43.09	-0.24	(-2.96, 2.48)	0.86

H19 CTCF6	65	38.37	28	37.48	1.38	(-2.89, 5.64)	0.53

IGF2DMR0	65	36.52	27	35.03	2.07	(-0.28, 4.42)	0.08

IGF2R	65	79.45	29	81.35	-2.79	(-5.74, 0.16)	0.06

a Number of subjects included in the analysis

b The reported mean is averaged over all CpG sites in the region of interest

c Adjusted for pyrosequencing run, child's age, child's birth weight, child's sex, maternal age, maternal education, and household income

In the subgroup analysis, couples with unexplained infertility had children that tended to have lower methylation levels compared with couples in which both partners had an identified cause of infertility (Table 3). Sample size was small, however, and no overall group comparison was statistically significant.

**Table 3:** Adjusted regression model results by infertility diagnosis

Gene region of interest	Female vs. Both^a^		Male vs. Both^a^		Unexp vs. Both^a^		Group comparison p-value
		
	Difference^b^	(95% CI)	Difference^b^	(95% CI)	Difference^b^	(95% CI)	
Lymphocyte samples							

KvDMR	0.03	(-1.75,1.81)	-0.21	(-2.84, 2.43)	-2.71	(-6.40, 0.98)	0.51

H19 CTCF3	-0.96	(-2.96, 1.05)	-1.10	(-3.26, 1.06)	-2.62	(-5.99, 0.76)	0.56

H19 CTCF6	-0.89	(-3.82, 2.04)	1.01	(-2.68, 4.71)	-1.98	(-8.61, 4.66)	0.72

IGF2DMR0	-1.26	(-3.24, 0.71)	-1.03	(-3.59, 1.53)	-3.45	(-6.87,-0.04)	0.40

IGF2R	0.43	(-4.02, 4.88)	-3.40	(-7.48, 0.67)	-5.92	(-13.06, 1.22)	0.39

Buccal samples							

KvDMR	-5.50	(-10.86,-0.15)	-4.03	(-9.42, 1.37)	-7.40	(-14.66,-0.15)	0.14

H19 CTCF3	-1.63	(-5.29, 2.02)	-1.84	(-5.95, 2.27)	-4.72	(-9.41,-0.03)	0.37

H19 CTCF6	-3.52	(-8.37, 1.33)	-0.77	(-7.57, 6.02)	-5.16	(-12.33, 2.00)	0.30

IGF2DMR0	0.17	(-2.86, 3.19)	0.57	(-2.64, 3.78)	-1.34	(-5.93, 3.26)	0.82

IGF2R	0.76	(-1.80, 3.32)	1.31	(-0.94, 3.55)	-3.64	(-10.24, 2.95)	0.54

a Infertility diagnoses were divided into the following groups: Female factors (Female) n = 21, Male factors (Male) n = 17, Both male and female factors (Both) n = 16, and Unexplained (Unexp) n = 6

b Adjusted for pyrosequencing run, child's age, child's birth weight, child's sex, maternal age, maternal education, and household income

## Discussion

Overall, there was little difference in methylation between children conceived through ART and children conceived spontaneously. In subgroup analysis, couples with unexplained infertility tended to have lower methylation levels compared to couples in which both partners had an identified cause of infertility.

The observed differences in methylation between the ART and SC group were very small, indicating that our failure to detect moderate differences was not due to a lack of statistical power even though variability was increased due to the small sample size and imperfect assay reliability [41]. Larger samples or samples with increased reliability could be used to detect smaller differences; however, we were able to rule out differences in methylation greater than about 7% in all analysis. For lymphocyte samples, average differences were around 1% or less between groups. Based on the variability of the samples, our analysis suggested that differences greater than 2-4 percentage points for all loci except *IGF2R *are unlikely. Methylation in buccal cell samples was more variable, with an average difference of around 2%. For these samples, differences greater than 5-7 percentage points appeared unlikely. It should be noted that it is unknown if very small differences in methylation can lead to difference in gene expression levels; so, although it seems unlikely that there is an effect for a few percentage points difference in methylation, it is possible. One study did find that small differences in DNA methylation resulted in differences in transcript levels, suggesting that these small differences could be biologically relevant [26].

Other studies have examined differential methylation in various tissues of children conceived through ART with normal phenotypes (Table 4). Small, non-significant differences between groups, such as those seen in our study, have been observed in many studies (Additional file 1). The most common region that has been examined is the *IGF2*/*H19 *imprinting region. In placenta tissue, two studies have indicated a potential difference in methylation and expression in *H19 *and *IGF2 *[33] and expression only in *H19 *but not *IGF2 *[30] while another study found no difference in methylation [34]. Two studies examined possible difference in this region in miscarriages, abortions, and stillbirths with one finding a possible difference but more extreme values in the control group [35] and the other finding six cases with hypomethylation in the ART group and none in the control group [27]. In addition, a study on embryos from ART patients found that close to 19% of these embryos had hypomethylation or demethylation in the *H19 DMR *[23]. Overall, there is some evidence for possible hypomethylation or reduced methylation in children conceived through ART in several studies [23,27,31,35]; however, the majority of studies have not found a statistically significant difference between groups [25,26,28,31,32,34,35,38].

**Table 4:** Summary of previous studies examining DNA methylation differences in ART conceptions

Author	Tissue	ART group	SC group	Gene/Region	Findings
Oliver, et al. [38]	Blood	66 (34 IVF, 32 ICSI)	59	*H19, KCNQ1OT1*, *SNRPN, IGF2*, *LOC388665, INSL5, ARHGAP24, STK19, NCRNA00282, JPH4*, *SYP, BEX1*	Only significant difference in *NCRNA00282 *and possibly *ARHGAP24*

Zheng, et al. [36]	Cord blood	101 (40 ICSI, 61 IVF)	60	*KvDMR1, SNRPN*, *MEST, MEG3, TNDM*, *XIST*	All showed normal methylation patterns

Zheng, et al. [37]	Chorionic villus samples	44 spontaneous abortions, 22 multifetal reductions	45 spontaneous abortions, 47 induced abortions	*PEG1/MEST*	Higher methylation in spontaneous abortions in ART or SC group compared with other two groups, no difference between ART and SC

Wong, et al. [34]	Placenta	77 (32 IVF, 45 ICSI)	12	*H19/IGF2 ICR1*	No difference between groups consisting of IVF, ICSI, SC stratified by size for gestational age, 6 ART and 2 SC cases with hypomethylation

Shi, et al. [31]	Cord blood	61	30	*IGF2/H19 ICR*	No difference between groups, but three ICSI children had abnormal demethylation

Li, et al. [28]	Cord blood	29 twin pairs	30 twin pairs	*KvDMR1, PEG1*, *H19/IGF2 DMR*	No significant differences, but lower methylation in *KvDMR1 *and higher methylation in *H19/IGF2 *in ART twins.

Zechner, et al. [35]	Chorionic villus samples	42 spontaneous miscarriages and stillbirths	29 abortions and stillbirths	*H19, MEG3, LIT1*, *MEST, NESP55, PEG3*, *SNRPN, NANOG, APC*	No significant difference between groups, more extreme methylation values in the control group

Turan, et al. [33]	Cord blood, cord, placenta	45	56	*IGF2/H19, IGF2R*	The maternal to paternal methylation ratio mean and variance were greater in the IVF group for both tissues (means in cord and placenta and variance in cord blood and cord), no differences for IGF2R

Tierling, et al. [32]	Maternal blood, cord blood, amnion/chorion tissue	112 (77 ICSI, 35 IVF)	73	*KvDMR1, H19*, *SNRPN, MEST, GRB10, DLK1/MEG3, Ig-DMR, GNAS NESP55, GNAS NESPas, GNAS XL-* *alpha-s, GNAS Exon1A*	Difference in *MEST *in cord blood (IVF vs. ICSI or IVF vs. SC), higher *MEST *methylation in IVF mothers vs ICSI or SC; also possible differences in *DLK1/MEG3 *and the *GNAS *region in cord blood

Chen, et al. [23]	Embryos	32	0	*H19 DMR*	18.7% with hypomethylation

Kobayashi, et al. [27]	Trophoblastic villi	78 abortions	38 non-ART abortions	*H19, GTL2, PEG1, KCNQ1OT1, ZAC*, *PEG3, SNRPN*	Hypomethylation in ART cases relative to controls for *H19 *(n = 6), *GTL2 *(n = 2), *PEG1*(n = 1), *LIT1* (n = 4), *ZAC *(n = 1), *PEG3 *(n = 1)

Katari, et al. [26]	Cord blood, placenta	10	13	Genome wide, 736 genes	Differentially methylated CpG sites tended to have higher methylation in cord blood and lower methylation in placenta

Kanber, et al. [25]	Buccal cells	19 small for gestational age and ICSI	29 normal birth weight	*KCNQ10T1, IGF2/H19, PEG1*, *PEG3, PLAGL1, GTL2*	1 ICSI child with hypermethylation of *KCNQ1OT1* and *PEG1*, 2 control children had hypermethylation of *GTL2*

Gomes, et al. [24]	Peripheral blood, Cord blood, placenta	12 peripheral blood, 6 cord blood, placenta	22 peripheral blood, 8 cord blood, placenta	*KvDMR1*	3 ART children with hypomethylation in blood, lower levels in ART vs. SC, but not statistically significant

Palermo, et al. [30]	Blood	55	0	*SNRPN*	No differences from expected

Manning, et al. [29]	Blood	92 ICSI	Reference sample	*SNRPN*	No differences from expected

For the *KvDMR *region, some expression differences have been seen in placenta for *CDKN1C*, but not *KCNQ1OT1 *[30]. Other studies have found no differences within this region [27,32,35,36], although one did note hypomethylation in three children conceived through ART [24] and another found hypermethylation of *KCNQ1OT1 *in a child conceived using ART [25]. As with the IGF2/H19 region while there is some evidence of possible hypomethylation [24,27,28,35], most studies have not found a statistically significant difference [24,26,28,32,36,38].

Two regions examined in this study are relatively novel, having only been examined in one prior study. The DMR in the *IGF2R *gene is of particular interest since it has not be well studied, and a recent study associated gain of methylation at this locus with growth restriction [42]. We found a non-significant decrease in methylation within the *IGF2R *region for both lymphocyte and buccal cell samples, which would be in contrast to the growth restriction study, since children conceived through ART tend to be smaller at birth [43]. The only other study examining this locus in children conceived through ART also did not find a difference in DNA methylation in cord blood, cord samples, or placenta, but quantitative results are difficult to compare since that study used the ratio of maternal to paternal allele methylation as their primary outcome [33]. The *IGF2 DMR0 *was only recently examined in a study by Oliver, et al., who found no difference in DNA methylation at this region in peripheral blood samples [38]. While our study concurs with these finding for peripheral blood, we did find non-significant increase in DNA methylation at this region in buccal cells of children conceived through ART, so small differences in DNA methylation cannot be ruled out for this locus.

Another study used a methylation bead-array platform and examined 1536 CpG sites in over 700 genes using placenta and cord blood samples and found an overall lower level of methylation at CpG sites in the placenta samples of *in vitro *conceived children and a higher level of methylation in cord blood of *in vitro *conceived children [26]. This study also found many individual genes that had differential methylation between the two groups. However, none of the genes analyzed in our study were specifically identified.

Other regions that have been explored in multiple studies include *SNRPN, MEG1/GTL2, PEG1/MEST*, and *PEG3*. No studies have found evidence of a difference in DNA methylation in the *SNRPN *region in children conceived through ART compared with an SC group [26,27,29,30,32,35,36,38]. Differences have also not been observed for the *MEG1/GTL2 *region in multiple studies [25,26,32,35,36]. Methylation differences were found between the ART and SC group in CpG sites associated with the *PEG1/MEST *region in both cord blood and placenta [26]. A second study support this finding, but also found differences in methylation in maternal blood, suggesting that the pattern seen in offspring was not due to the infertility treatment [32]. Other studies have not found a quantitative difference [28,35,37]; however one study found a case of hypermethylation in buccal cells [25] and one found a case of hypomethylation in extra-embryonic tissue [27]. *PEG3 *was also identified as have different methylation patterns between the ART and SC group from the genome-wide study both in cord blood and placenta [26], but other studies have not confirmed this finding [25,35]. One study found evidence of hypomethylation in one ART case in this region [27]. Other regions have been identified in single studies, but have not been confirmed.

The current study adds to the growing body of literature examining methylation differences in non-syndromic children conceived through ART and children conceived spontaneously. This study explored several specific loci associated with growth, cancer, and BWS some of which have been rarely studied in this population. Using a quantitative measure of DNA methylation is sensitive to detect subtle differences between groups which may influence transcription and gene expression [26]. It also included two sample types easily available after birth to permit broad follow-up studies. Finally, even though our sample size was not large, it is unlikely that we missed substantial differences in methylation based on our confidence intervals.

There are several caveats that should be addressed in this study. First, methylation abnormalities such as the type we are looking for here could be tissue specific rather than a global phenomenon. While it would be interesting to examine other tissues, it would not be ethical to obtain other tissue/sample types in otherwise healthy children since most collection procedures would be invasive. Unexposed individuals were a convenience sample rather than a random sample from a particular population. Women who use ART are more likely to be white, have higher incomes, and be better educated compared to infertile women who choose other or no treatment [44]. Although we observed some demographic differences between the ART and SC groups, mothers in the SC group were more likely to be white and have higher income and education levels compared to the US population and thus may represent a good comparison group. Finally, a limited number of genetic loci were evaluated in this study. Only those with the most *a priori *likelihood of an association were examined to maximize the potential for finding important associations.

## Conclusions

Overall, very small differences were observed in methylation level at all loci between groups. Some possible but non-significant differences in buccal cells and possible differences by infertility diagnosis may warrant additional follow up in other studies. The bulk of studies performed so far indicate that large differences in methylation are unlikely. However, it is not known the potential biological effect that small differences in methylation could have and there is some evidence that these might be relevant. Although results of this study are reassuring, additional large studies that include a broad range of genes as well as long term follow-up of children conceived through ART are needed to fully assess possible epigenetic differences and the potential impact of small differences on future disease in this population.

## Abbreviations

AS: Angelman syndrome; ART: Assisted reproductive technology; BWS: Beckwith-Wiedemann syndrome; CI: Confidence interval; GEE: Generalized estimating equations; ICC: Intraclass correlation coefficient; ICSI: Intracytoplasmic sperm injection; IQR: Interquartile range; IVF: *In vitro *fertilization; SRS: Silver-Russell syndrome; SC: Spontaneously conceived; US: United States.

## Competing interests

The authors declare that they have no competing interests.

## Authors' contributions

SEP designed the study, directed its implementation, performed the study analysis, and prepared and revised the text. HHN participated in the study design, supervised the DNA methylation analysis, and critically reviewed and revised the text. JAR participated in the study design, supervised sample preparation and DNA extraction, and critically reviewed and revised the text. RHNN assisted in the study design and critically reviewed and revised the text. MAD assisted in case enrollment and critically reviewed and revised the text. LGS assisted in the study design and implementation and helped prepare and revise all sections of the text. All authors read and approved the final manuscript.

## Pre-publication history

The pre-publication history for this paper can be accessed here:

http://www.biomedcentral.com/1471-2431/12/33/prepub

## Supplementary Material

Additional file 2 **Intraclass correlation coefficients for individual CpG sites**. Description: Table of the intraclass correlation coefficients for the reliability analysis for the individual CpG sites. Click here for file

Additional file 1 **Detailed summary of the literature on DNA methylation in children after ART**. Two tables are included providing qualitative (hypomethylation or hypermethyation) and quantitative methylation differences between children conceived through ART and those conceived spontaneously. One table presents the data for studies examining blood or buccal cells and the other presents the same data for extra-embryonic tissues. Click here for file
